# Factors associated with study completion in patients with premature acute coronary syndrome

**DOI:** 10.1371/journal.pone.0173594

**Published:** 2017-03-16

**Authors:** Anthony W. Austin, Roxanne Pelletier, Louise Pilote, Doreen M. Rabi, for the GENESIS-PRAXY Investigators

**Affiliations:** 1 Department of Social and Behavioral Sciences, University of Arkansas at Pine Bluff, Pine Bluff, Arkansas, United States of America; 2 Centre de psychologie Au fil des maux, Mont-Saint-Hilaire, Quebec, Canada; 3 Divisions of General Internal Medicine and Clinical Epidemiology, McGill University Health Centre, Montréal, Quebec, Canada; 4 Departments of Medicine, Community Health and Cardiac Sciences, University of Calgary, Calgary, Alberta, Canada; JAPAN

## Abstract

**Background:**

Factors associated with study completion in younger adults are not well understood. This study sought to describe psychosocial, clinical, and demographic features associated with completion of a study of men and women with premature acute coronary syndrome.

**Methods:**

As part of the GENdEr and Sex determInantS of cardiovascular disease: From bench to beyond-Premature Acute Coronary Syndrome (GENESIS-PRAXY) study, demographic, psychosocial, and clinical variables were assessed in 1213 patients hospitalized for acute coronary syndrome (≤ 55 years; 30% women). Patients were followed for 12 months. Dropouts withdrew from the study or were lost to follow-up after 12 months; completers were still enrolled after 12 months.

**Results:**

Of 1213 patients initially enrolled, 777 (64.1%) completed 12-month follow-up. Fully adjusted models suggested that being older (OR = 1.04, 95% CI [1.01, 1.06]), higher subjective social status within one’s country (OR = 1.11, 95% CI [1.01, 1.22]), being free of type II diabetes, (OR = 0.66, 95% CI [0.45, 0.97]), non-smoking status (OR = 0.70, 95% CI [0.51, 0.95]) and being free of depression (OR = 1.52, 95% CI [1.11, 2.07]) were independently associated with study completion.

**Conclusions:**

Recruitment/retention strategies targeting individuals who smoke, are younger, have low subjective social status within one’s country, have diabetes, or have depression may improve participant follow-up in cardiovascular cohort studies.

## Introduction

Ensuring participation and retention of subjects in cohort studies remains a challenge, as rates of attrition in cohort studies have increased over the past several decades [1–3]. When data are missing completely at random, measures of association and inferences drawn from the results are typically valid. However, attrition is rarely a completely random event in cohort studies [4]. More likely the case, those who complete cohort studies (“completers”) differ systematically from those who are lost to follow up (“dropouts”), which in turn reduces the reliability and generalizability of the results and lowers statistical power [4–6].

In general, several factors have univariate associations with not completing longitudinal health studies, including being older, being cognitively impaired, increasingly ill health, living alone, not being married, lower socio-economic status (SES), less social activity, and obesity. Conversely, being retired and having more living children are associated with greater study completion [7, 8]. However, only increasing age and cognitive impairment seem to be independently associated with not completing longitudinal studies [8]. Moreover, few studies have examined whether completers and dropouts differ systematically in cohorts of cardiovascular patients or cardiovascular prevention [9–11]. In these studies, study completion was associated with several sociodemographic (i.e., higher income, lower body mass index (BMI), older age, female sex, and white ethnicity), access-to-care (i.e., attendance of health fairs and having health insurance), clinical (i.e., not smoking, having metabolic syndrome and hypertension, lower HDL cholesterol level, and adherence to cardiac medication), and psychosocial (i.e., lower levels of depression, anxiety, and general stress) characteristics [9–11]. These studies, however, had important limitations. For instance, one study only reported unadjusted results [9] whereas another did not examine men or report their female participants’ age [11]. Moreover, the two studies that did report age [9, 10] only examined older adults (i.e., greater than 59 years). However, no known study has examined predictors of study completion in younger patients with premature acute coronary syndrome (ACS). It is important to examine such younger individuals because they likely have a different life situation than older adults, e.g., work, child rearing, and income, and these factors may influence study completion differently.

Identifying factors associated with study completion in cardiovascular cohorts is a step toward developing and/or improving recruitment and retention strategies. Therefore, the purpose of this study was to identify factors associated with completion of a longitudinal study of patients with premature ACS.

## Materials and methods

### Study design and protocol

We report a sub-analysis of the GENdEr and Sex determInantS of cardiovascular disease: from bench to beyond—PRemature Acute Coronary SYndrome (GENESIS-PRAXY) study. This is a prospective, multicenter study with a 12-month follow-up of patients with premature ACS. Patients were recruited from 24 centers in Canada, one in Switzerland, and one in the United States, from January 2009 to April 2013. A research nurse contacted the patients by telephone at 1, 6, and 12 months. At these times, the nurse validated the participants’ addresses and then mailed them questionnaires to complete and return. Full details of the methods have been explained previously [12]. Approval of the study was obtained from all sites from their respective ethics review boards. Overall approval of the study was granted by the University of British Columbia Providence Health Care Research Institute (Reference number: PHC REB H08-01382). Additionally, ethics approval was received from the ethics committees of the following institutions: St Paul's Hospital, Vancouver, British Columbia, Canada; Surrey Memorial Hospital, Surrey, British Columbia, Canada; Libin Cardiovascular Institute of Alberta, University of Calgary, Calgary, Alberta, Canada; University of Alberta and the Mazankowski Alberta Heart Institute, Edmonton, Alberta, Canada; University of Ottawa Heart Institute, Ottawa, Ontario, Canada; McMaster University/Hamilton Health Sciences (General Site), Hamilton, Ontario, Canada; McMaster University/Hamilton Health Sciences (Juravinski Site), Hamilton, Ontario, Canada; Ottawa Hospital, Ottawa, Ontario, Canada; St Michael's Hospital, Toronto, Ontario, Canada; London Health Sciences Centre, London, Ontario, Canada; The Scarborough Hospital, General Division, Scarborough, Ontario, Canada; Hôpital Général de Montréal, Montréal, Québec, Canada; Hôpital Royal Victoria, Montréal, Québec, Canada; Hôpital Général Juif-Sir Mortimer B. Davis, Montréal, Québec, Canada; Institut universitaire de cardiologie et de pneumologie de Québec (Hôpital Laval), Québec, Québec, Canada; Hôpital du Sacré-Coeur de Montréal, Montréal, Québec, Canada; Cité de la Santé de Laval, Laval, Québec, Canada; Hôtel Dieu du Centre Hospitalier de l'Université de Montréal, Montréal, Québec, Canada; Centre de santé et de services sociaux de la région de Thetford, Thetford Mines, Québec, Canada; CSSS Chicoutimi, Chicoutimi, Québec, Canada; Centre Hospitalier Universitaire de Sherbrooke, Sherbrooke, Québec, Canada; CSSS Alphonse Desjardins (CHAU—Hôtel-Dieu de Lévis), Lévis, Québec, Canada; Queen Elizabeth II Health Science Centre, Halifax, Nova Scotia, Canada; The New Brunswick Heart Centre Research Initiative and The New Brunswick Heart Centre, New Brunswick, Canada; Basset Healthcare, Cooperstown, New York, USA; Inselspital, University of Bern, Switzerland and Lausanne University Hospital, Lausanne, Switzerland.

Written informed consent was obtained from all participants and the study was carried out in accordance with the Declaration of Helsinki.

### Study completion

To augment completion of questionnaires at each follow-up, the following procedure was performed: 1) A research nurse called the patient a few days before the date of the follow-up to ensure the patient’s address was correct and to remind the patient that they will receive a questionnaire by mail, which they will need to complete and return in the stamped envelope. 2) Once the address was confirmed, the nurse sent the questionnaire by mail. 3) If the address could not be confirmed, the nurse would send the questionnaire to the address indicated in the patient’s file. 4) If the questionnaire was not received within a few weeks, the nurse repeated steps 1–3 up to two additional times. 5) If preferred, participants could complete the questionnaire over the phone. This occurred only in 8 patients. 6) If questionnaires were received at all three follow-ups, participants received a $20 gift certificate.

Participants who could not be contacted or did not return the questionnaires at 12 months for any reason other than death were classified as dropouts. Those who completed the questionnaires at 12 months were classified as completers.

### Study population and data sources

Patients presenting to a participating hospital with ACS who were between 18 and 55 years old, fluent in French or English, and capable of giving informed consent were eligible. A research nurse approached each eligible patient in the coronary care unit as soon as possible after hospital admission. A total of 1213 patients were recruited.

### Potential factors associated with study completion

Based on previous research [7–11], several characteristics were identified as potential factors associated with study completion. Medication use at time of hospitalization (i.e., Calcium antagonists, ACE inhibitors, and statins), number of medications at time of discharge, previous diagnosis of dyslipidemia, hypertension, and diabetes were determined from chart review. Body mass index was calculated from height and weight. From self-report data, age, sex, ethnicity, education level, work status, job satisfaction, income, smoking status, availability of health insurance, access to health care, satisfaction with health care, stress at work, stress at home, and overall stress level were determined. “Satisfaction with health care” variables were assessed with one question each. Availability of health care was assessed with the question “Overall, how would you rate the availability of health care services in your area?” Quality of health care was assessed with the question “Overall, how would you rate the quality of the health care services that are available in your area?” Participants answered these two questions on a 1–4 scale with 1 = Poor and 4 = Excellent. Satisfaction with treatment was assessed with the question “Overall, how satisfied are you with the treatment that you received, over the past 12 months?” Participants answered this question on a 1–5 scale with 1 = not satisfied at all and 5 = very satisfied. Stress at work, stress at home, and overall stress level were measured on a scale from 1 (no stress) to 10 (most stress).

Based on items in our questionnaire, major depression was assessed using the Diagnostic and Statistical Manual of Mental Disorders, 4^th^ edition criteria [13]. Specifically, participants were asked to think about how they felt prior to their ACS and answer nine questions representing DSM-IV criteria for major depression. Major depression was subsequently identified if five or more of the nine questions were answered “yes” and if one of the symptoms was either a loss of interest in daily activities or feeling blue sad, or depressed most days of the week for two weeks or more in a row [13]. Symptoms of depression and anxiety were also assessed with the Hospital Anxiety and Depression Scale [14].

Traits of personality traditionally ascribed to men and women were assessed with the Bem Sex Role Inventory [15], which produces independent scores of “femininity” and “masculinity”. Based on gender stereotypes, it measures the extent to which a person identifies as fitting into traditional sex roles. A high masculinity score does not necessarily indicate a low femininity score, and vice versa, because masculinity scores and femininity scores are mutually exclusive. Subjective social status within one’s community and within one’s country was assessed with the MacArthur Subjective Social Status Scale (MSSS) [16]. Independent of objective measures of socioeconomic status like income and education, the MSSS aims to denote respondents’ view of where they stand in society, considering multiple dimensions of socioeconomic status and social position. For income, we compared those who reported being below low-income cutoffs to those above the cutoffs, based on Stats Canada figures [17]. Specifically, we compared those who reported personal income to be $22,500 or less to those who reported making more than $22,500.

### Statistical analyses

Baseline characteristics were compared between the completers and dropouts. Continuous variables were compared using t-tests or Wilcoxon tests, whereas categorical variables were compared using χ^2^ tests. Those variables in which completers and dropouts differed statistically significantly were further included in a stepwise manner in four multivariable logistic regression models. For these models, BMI was examined as not obese (BMI < 30) versus obese (BMI ≥ 30). The first model included sociodemographic variables. The second model included sociodemographic and access to care variables. The third model included sociodemographic, access-to-care, and clinical variables. The fourth model included sociodemographic, access to care, clinical, and psychosocial variables. All analyses were performed using SAS statistical package version 9.2 (Cary, North Carolina). Two-tailed p-values < .05 were considered significant.

## Results

### Participant characteristics and univariate analyses

Of the 1213 patients initially enrolled, 777 (64.1%) completed the study through 12 months follow-up. In comparison to those in the dropouts group, those in the completers group were older, had a lower Bem masculinity score, greater subjective social status within their community and within their country, and greater satisfaction with availability of health care. Moreover, completers were more likely to be white compared to other ethnicities, be working, be over the poverty line, have a diploma or degree, have a family doctor, be nonsmokers, not have type II diabetes, not have major depression and report experiencing more stress at work (Table 1).

**Table 1:** Sociodemographic, clinical, access to care, and psychosocial characteristics by categories of 12-month retention.

	Dropouts group		Completers group		p
Sociodemographic variables	n	Mean/Median	n	Mean/Median	
**Age (yrs), median (IQR)**	433	49 (8.00)	773	50 (7.00)	.0351*
**Sex, n (% female)**	435	139 (31.95)	777	253 (32.56)	.8284
**Bem score, mean (SD)**					
Femininity score	374	5.71 (0.98)	756	5.63 (0.91)	.1653
Masculinity score	375	5.06 (0.94)	755	4.93 (0.91)	.0301*
**Ethnicity, n (% not white)**	435	99 (22.81)	777	107 (13.77)	< .0001^Ψ^
**Subjective Social Status, mean (SD)**					
In the community	358	5.74 (2.07)	745	6.06 (1.96)	.0129*
In one’s country	358	5.10 (2.14)	742	5.67 (2.05)	< .0001^Ψ^
**Currently working, n (%)**	435	291 (66.89)	777	612 (78.76)	< .0001^Ψ^
**Job Satisfaction, mean (SD)**	307	62.36 (15.00)	641	62.78 (13.89)	.6749
**Personal annual income, n (% < $22,500)**	317	135 (42.59)	651	207 (31.8)	.001**
**Education, n (% with post-secondary degree)**	328	83 (25.30)	663	260 (39.22)	< .0001^Ψ^
**Access to Care variables**					
**Receive supplementary medical benefits, n (%)**	262	204 (77.86)	613	478 (77.98)	.9701
**Have a family doctor, n (%)**	375	297 (79.20)	761	646 (84.89)	.0164*
**Difficulty getting routine health care, n (%)**	359	73 (20.33)	709	140 (19.75)	.8202
**Difficulty getting specialist health care, n (%)**	284	48 (16.90)	584	86 (14.73)	.4053
**Satisfaction with health services, mean (SD)**					
Availability of health care	368	2.16 (0.96)	748	1.99 (0.86)	.0025**
Quality of health care	367	1.93 (0.87)	743	1.86 (0.81)	.1533
Satisfaction with treatment	351	3.96 (1.06)	738	4.07 (1.01)	.0897
**Clinical Characteristics**					
**Smoking, n (%)**	434	187 (43.09)	777	275 (35.39)	.0082**
**Taking Cardiovascular Medications at time of hospitalization, n (%)**					
Calcium antagonists	434	27 (6.22)	777	50 (6.44)	.8837
ACE inhibitors	423	300 (70.92)	762	542 (71.13)	.9401
Statins	433	400 (92.38)	777	712 (91.63)	.6492
**Number of drugs at discharge, (SD)**	433	6.11 (2.24)	777	5.98 (3.62)	.5171
**Obese, n (%)**	424	190 (44.81)	761	295 (38.76)	.0424*
**Hypertension, n (%)**	435	207 (47.59)	777	377 (48.52)	.7550
**Type II Diabetes, n (%)**	435	94 (21.61)	777	114 (14.67)	.0021**
**Dyslipidemia, n (%)**	434	234 (53.92)	777	434 (55.86)	.5153
**Psychosocial characteristics**					
**HADS score**					
Depression subscale, n (% ≥ 8)	435	110 (25.29)	777	162 (20.85)	.0757
Anxiety subscale, n (% ≥ 8)	435	184 (42.30)	777	301 (38.74)	.2249
**Major Depression**	435	120 (27.59)	777	165 (21.24)	.0124*
**Stress**					
Stress at work, n (% ≥ 4)	435	210 (48.28)	777	424 (54.57)	.0354*
Stress at home, n (% ≥ 4)	435	135 (31.03)	777	238 (30.63)	.8838
Overall stress, n (% ≥ 4)	435	192 (44.14)	777	342 (44.02)	.9671

*Note*.

* p < .05,

** p < .01

*** p < .001,

^Ψ^ p < .0001.

Satisfaction with health care variables are rated on a scale from 1–4.

### Multivariate analyses

**Fig 1:**
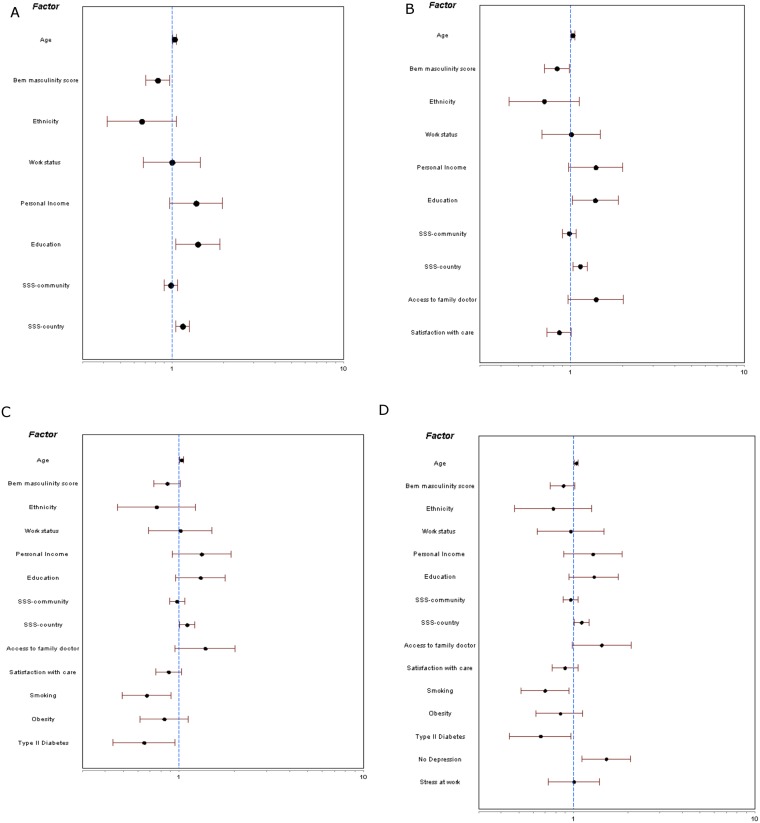
Forest plots of adjusted associations of sociodemographic, access to care, clinical, and psychosocial factors on study completion. a) Adjusted for sociodemographic factors alone; b) Adjusted for sociodemographic and access to care factors; c) Adjusted for sociodemographic, access to care, and clinical factors; d) Fully adjusted for sociodemographic, access to care, clinical, and psychosocial factors.

Variables having univariate associations with study completion were included in the multivariate analyses (Fig 1). In the first logistic regression model including sociodemographic variables, older age (OR = 1.04, 95% CI [1.01, 1.06]), a lower BEM masculinity score (OR = 0.83, 95% CI [0.70, 0.97]), having a diploma or degree (OR = 1.41, 95% CI [1.05, 1.91]), and higher subjective social status within one’s country (OR = 1.16, 95% CI [1.05, 1.27]) were associated with greater odds of study completion. When adding access to care variables in the second model, older age (OR = 1.03, 95% CI [1.01, 1.06]), lower BEM masculinity score (OR = .84, 95% CI [.71, .98]), having a diploma or degree (OR = 1.39, 95% CI [1.03, 1.88]) and higher subjective social status within one’s country (OR = 1.14, 95% CI [1.04, 1.25] were still associated with greater odds of study completion, whereas access to care variables were not.

When adding clinical characteristics in the third model, older age (OR = 1.04, 95% CI [1.01, 1.06]), and higher subjective social status within one’s country (OR = 1.11, 95% CI [1.01, 1.22]) remained associated with greater odds of study completion, as was being a nonsmoker (OR = 0.67, 95% CI [0.49, 0.91], and not having Type II diabetes (OR = 0.65, 95% CI [0.44, 0.96]). Education was no longer associated with greater odds of study completion. When including psychosocial variables in the fourth and fully adjusted model, older age (OR = 1.04, 95% CI [1.01, 1.06]), higher subjective social status within one’s country (OR = 1.11, 95% CI [1.01, 1.22], not smoking (OR = 0.70, 95% CI [0.51, 0.95]), not having type II diabetes, (OR = 0.66, 95% CI [0.45, 0.97]), and not having depression (OR = 1.52, 95% CI [1.11, 2.07]) were associated with greater odds of study completion.

## Discussion

The GENESIS-PRAXY study enrolled 1213 patients with premature ACS. Over 12 months of follow-up, we found that older age and higher subjective social status within one’s country at baseline were independently associated with greater odds of study completion, whereas smoking, having type II diabetes, and depression at baseline were independently associated with lower odds of study completion.

Our age, smoking, and depression findings corroborate previous work in cardiovascular cohorts [9–11], suggesting that study completion may occur systematically depending on these patient characteristics. Younger individuals may feel less vulnerable to disease or less benefit from long-term involvement in research studies [10]. Those who smoke or have depression may be less likely to engage in health-focused interventions in general [18, 19] and lack of study completion may be an extension of this. Additionally, smoking and depression likely put individuals at greater risk of recurrence of coronary events. If patients in our study had a recurrent event during our study period, then this is one possible reason for not completing the study.

Identifying characteristics of study completion at study entry has important implications for retention in similar studies. For instance, it may be important to refer depressed patients for treatment when enrolling them in longitudinal case only studies. Lack of completion of health studies in depressed patients could be due to lack of motivation. Therefore, retention approaches specific to depressed patients may be worthwhile, e.g., collaborative care and motivational interviewing [20]. Similarly, retention strategies might need to address possible comorbid conditions such as type II diabetes that may prevent commitment to long-term study participation. We know of no other study that has found that type II diabetes independently predicts study completion in cardiovascular cohorts. However, type II diabetes was significantly associated with lower odds of study completion in univariate analyses in one study, though it was not significant in multivariate analyses [10]. Conversely, our results contradict another study in which metabolic syndrome was independently associated with greater odds of completion of cardiovascular community health interventions for women [11]. The authors of that study speculated that women with high-risk characteristics (e.g., metabolic syndrome) may feel more motivated to willingly engage in cardiovascular health studies in order to improve cardiovascular health. However, it may be more likely that those with fully developed type II diabetes may feel that participating in cardiovascular health studies may not benefit them. Finally, similar to smoking and depression, having type II diabetes may put one at greater risk of a recurrent coronary event, which in turn may influence study completion.

This study had noteworthy strengths. First, this is the first study to assess predictors of study completion in a cohort of premature ACS. Second, improving on the sole inclusion of income and education, the subjective social status measures give a fuller picture of the role socioeconomic status plays in study completion in longitudinal studies, with implications across disciplines. Third, we included a considerable proportion of women (30%). It is difficult to recruit young women with ACS, given that premature ACS occurs most frequently in men. Finally, our large sample size and multicenter design suggests that our results are generalizable across geographic regions, especially in Canada.

An important implication of our depression finding is that the association between depression and cardiovascular outcomes may be underestimated if the most depressed do not complete studies such as ours. Therefore, retention strategies targeting depressed individuals may need to be developed.

A novel finding of our study was that increased subjective social status within one’s country was independently associated with greater odds of study completion, whereas objective measures of socioeconomic status such as income, education, and working status were not. Income is only one component of subjective social status, which may explain why income was not an independent predictor of study attrition in our study but was previously reported to be [10]. Those with lower subjective social status tend to report less optimism, less control of life, and more chronic stress than those of higher subjective social status [21, 22]. Together, these traits may lead to less ability to complete longitudinal studies. Additionally, those who have lower subjective social status may not perceive benefits to themselves or to society when participating in health studies and therefore do not complete all phases of a longitudinal study. With implications for all participants, but especially for depressed individuals and those who perceive their social position to be low, researchers should also work to dissipate the belief that they are more interested in research than they are in patient’s well-being [10].

### Limitations of this study

Some limitations of our study must be addressed. First, the average income in Switzerland is much higher (median disposable income = 65,800 USD) than in Canada (median disposable income = 36,500 USD) and the United States (median disposable income = 30,900 USD) [23], potentially skewing our results. That is, there was only a very small portion of Swiss participants with income less than 22,500 (n = 56, 4.62%). However, using the MSSS mitigates this limitation by assessing relative social status in addition to absolute income, which may vary greatly. Second, incomplete data at baseline precluded using the full sample in the analyses. This may reduce the reliability of our findings. Third, our results may not be generalizable to other cardiovascular cohorts, i.e., they are specific to younger patients with ACS over 12 months follow-up. For instance, studies with older cardiovascular cohorts reported that greater income, having hypertension, female sex, and having health insurance were associated with study completion [10, 11]. However, these divergent findings may be due to methodological differences. We examined more variables and some of the significant findings in previous studies may have been rendered nonsignificant, had they adjusted for more factors. Third, our analyses pertaining to ethnicity were limited to white versus non-white. In order to examine ethnicity, we had to collapse non-white individuals into one group because of the small numbers within each non-white ethnicity. Finally, shorter and longer term studies may have different determinants of study completion. Our follow-up was relatively short (1 year). A cardiovascular prevention study with a 4-year follow-up reported that lower BMI, female sex, older age, and having health insurance were predictors of study completion, in addition to depression [10]. Had we followed patients for a longer period of time, our results may have mirrored theirs.

### Conclusions

We identified higher subjective social status, not smoking, older age, no type II diabetes, and no depression at baseline as independent predictors of study completion. Our results may be used to further develop awareness and appreciation of biopsychosocial obstacles that lie in the path to participating in longitudinal CVD studies. Knowledge of these characteristics may be used to inform focused efforts to recruit and retain participants in future cohort studies.
